# From theory to practice: translating the concept of cognitive resilience to novel therapeutic targets that maintain cognition in aging adults

**DOI:** 10.3389/fnagi.2023.1303912

**Published:** 2024-01-12

**Authors:** Andrea R. Zammit, David A. Bennett, Aron S. Buchman

**Affiliations:** ^1^Rush Alzheimer’s Disease Center, Rush University Medical Center, Chicago, IL, United States; ^2^Department of Psychiatry and Behavioral Sciences, Rush University Medical Center, Chicago, IL, United States; ^3^Department of Neurological Sciences, Rush University Medical Center, Chicago, IL, United States

**Keywords:** cognitive resilience, cognitive aging, cognitive decline, neuropathology, molecular mechanisms

## Abstract

While the concept of cognitive resilience is well-established it has not been defined in a way that can be measured. This has been an impediment to studying its underlying biology and to developing instruments for its clinical assessment. This perspective highlights recent work that has quantified the expression of cortical proteins associated with cognitive resilience, thus facilitating studies of its complex underlying biology and the full range of its clinical effects in aging adults. These initial studies provide empirical support for the conceptualization of resilience as a continuum. Like other conventional risk factors, some individuals manifest higher-than-average cognitive resilience and other individuals manifest lower-than-average cognitive resilience. These novel approaches for advancing studies of cognitive resilience can be generalized to other aging phenotypes and can set the stage for the development of clinical tools that might have the potential to measure other mechanisms of resilience in aging adults. These advances also have the potential to catalyze a complementary therapeutic approach that focuses on augmenting resilience via lifestyle changes or therapies targeting its underlying molecular mechanisms to maintain cognition and brain health even in the presence of untreatable stressors like brain pathologies that accumulate in aging adults.

## Introduction

Aging is characterized by continuous changes in cognitive abilities that in many older adults can become prominent enough to warrant a diagnosis of mild cognitive impairment or Alzheimer’s disease (AD) dementia. Aging is also characterized by underlying and insidious accumulation of multiple brain pathologies and degeneration of neural structures crucial for cognition. Yet, not all individuals with evidence of accumulated brain pathologies and neurodegenerative changes manifest cognitive impairment or decline. Up to a third of individuals in the Religious Orders Study (ROS) and Rush Memory and Aging Project (MAP) cohorts (described in further detail below), who meet the criteria for a diagnosis of pathologic AD at autopsy do not manifest clinical signs of impaired cognition prior to death (Bennett et al., 2006a,b). Other reports have shown similar results (Crystal et al., 1988; Katzman et al., 1988; Snowdon, 1997). These studies led to the hypothesis that individuals who remain cognitively intact despite the presence of significant neuropathologic AD burden harbor resilience factors that offset the negative cognitive effects of accumulating brain pathologies (Mortimer, 1997; Cummings et al., 1998; Stern, 2002). While early clinical-pathologic studies (Crystal et al., 1988; Katzman et al., 1988; Snowdon, 1997), and later, neuroimaging studies (Reuter-Lorenz, 2002; Stern et al., 2003; Stern, 2017), provided further evidence supporting the cognitive resilience hypothesis, a lack of consensus on how the construct is operationalized has also prevailed.

### The concept of cognitive resilience has been difficult to operationalize

Cognitive resilience has been broadly conceptualized as the ability to maintain function despite physiologic or pathologic, stressors (Luthar et al., 2000; Wagnild and Collins, 2009; Windle, 2011; Fletcher and Sarkar, 2013; Jain et al., 2014; Whitson et al., 2016). Some individuals function better (or worse) than average vis-a-vis specific stressors. Prevailing perspectives have proposed various operational definitions to distinguish among different forms of resilience (Stern, 2009, 2012; Cabeza et al., 2018; Stern et al., 2019, 2020), yet they have only focused on protective factors that afford a beneficial effect on an individual’s function. Table 1 provides a list of current terminologies and definitions from prevailing frameworks (Reuter-Lorenz and Park, 2014; Arenaza-Urquijo and Vemuri, 2018; Stern et al., 2020; Abadir et al., 2023; Office of Dietary Supplements NIoH, Trans-NIH Resilience Working Group, 2023). Most of these terms are related, and the literature is ripe with studies teasing out proxies of reserve and applying various brain modalities to study what makes individuals (more) resilient to disease (Nyberg et al., 2012; Habeck et al., 2017; Anatürk et al., 2021; Vaqué-Alcázar et al., 2021; Gazes et al., 2023; Neth et al., 2023). Thus, while a wide range of potential reasons for why some individuals function better than average have been suggested, explanations on why other individuals might function worse than average have not been addressed. These overarching definitions acknowledge the multidimensionality of resilience and give value to various approaches to elucidate the mechanisms of resilience; however, the concept of resilience has remained a challenge to translate it into a tool that quantifies resilience on a person-specific level with aims to identify individuals with high or low resilience.

**Table 1:** List of current terminologies and definitions from prevailing frameworks on cognitive reserve and resilience.

Framework	Term	Definition
Reserve and Resilience Framework (Blanchard et al., 1996)	Cognitive reserve	Better-than-expected cognitive performance given the degree of brain aging, injury, or disease.
	Brain reserve	The neurobiological status of the brain at any point in time, e.g., neocortical size.
	Brain maintenance	The preservation of brain morphology, i.e., absence of neuropathologic changes.
Resilience and Resistance Framework (Arenaza-Urquijo and Vemuri, 2018)	Brain resilience	Better-than-expected cognitive performance in the presence of Alzheimer’s disease pathology. (This is similar to *cognitive reserve*).
	Brain resistance	The absence or lower-than-expect levels of pathology (This is similar to *brain maintenance*)
Scaffolding Theory of Aging and Cognition (Reuter-Lorenz and Park, 2014)	Compensatory scaffolding	The effects of neural processes that reduce the negative impact of brain aging on cognition.
National Institutes of Aging and the American Geriatrics Society (Abadir et al., 2023)	Resilience	A desirable outcome after exposure to a stressor that is expected to diminish the outcome.
Trans National Institutes of Health Resilience Working Group (Office of Dietary Supplements NIoH, Trans-NIH Resilience Working Group, 2023)	Resilience	Any system’s (individual, community, environment) capacity to resist, recover, grow, or adapt in response to a challenge or stressor.

This perspective will first give a brief overview of our prior clinical-pathologic work that has laid the foundations of our more recent work using genomic data to elucidate the neurobiological mechanisms of resilience. This work has led to approaches that seek to translate our findings into a qualifiable tool that can be used to measure the identified resilience mechanisms on a person-specific level. We treat cognitive resilience like most risk factors that lie on a wide continuum ranging from beneficial to detrimental, hence a population’s levels of resilience may span from low to average to high, again like any continuous risk factor. This approach allows us to utilize (and eventually quantify) the full spectrum of functioning (better, *or worse* than expected) given the exposure (neuropathologic burden), thus offering a more intuitive approach than *a priori* categories of yes/no resilience. We discuss in some detail how we approach the study of cognitive resilience with the aim of filling outstanding gaps in currently proposed frameworks.

### Our team initiated clinical-pathologic cohort studies to investigate cognitive resilience

The Religious Orders Study (ROS) and Rush Memory and Aging Project (MAP) began in 1994 and 1997, respectively, (together referred to as ROSMAP) to study aging and Alzheimer’s disease. The MAP was initiated as a complementary cohort study and extension of ROS, with the aims of identifying the structural basis of neural reserve, and of elucidating what determines its capacity. While ROS participants are Catholic nuns, priests, and brothers from more than 40 US groups, MAP participants are recruited from retirement communities and subsidized senior housing facilities throughout Chicago and Eastern Illinois. Participants are over the age of 65, enroll without known dementia, agree to annual clinical evaluations, cognitive testing, and blood draws, and agree to organ donation (Bennett et al., 2005, 2012, 2018). Both studies are ongoing.

Since the inception of these studies, we have conceptualized resilience via two basic mechanisms: one that degrades resilience and one that maintains it. Over the past two decades, we have provided evidence for both. First, we have shown that mechanisms that degrade resilience are not confined to AD pathology but include multiple pathologies including cerebral infarctions and Lewy bodies (Schneider et al., 2007a,b). Over the years our findings have also indicated that there are yet undiscovered neurobiological mechanisms that interact and damage neural networks that reduces the brain’s ability to tolerate these insults. For instance, psychosocial risk factors, such as chronic distress, loneliness, and higher reports of depressive symptomatology, have been associated with higher odds of dementia but were not associated with pathologic AD, Lewy bodies, or infarcts (Wilson et al., 2003, 2006, 2007a,b), indicating that other mechanisms that degrade resilience via these risk factors await discovery.

We have also shown that several psychosocial factors reduce the likelihood of clinical expression of brain pathologies by increasing or maintaining resilience mechanisms. For instance, we have reported on protective factors, such as education (Bennett et al., 2003), participation in cognitive activities (Wilson et al., 2002), social network size (Bennett et al., 2006c), cognitive processing (Boyle et al., 2008), purpose in life (Boyle et al., 2012), and physical activity (Buchman et al., 2019) that contribute to resilience by modifying the association between neuropathology and cognitive decline, i.e., these factors reduce the untoward effects of pathology on cognition. These findings are in line with the hypothesis that compensatory mechanisms in the nervous system might work more efficiently to circumvent insults, thus allowing the brain to maintain function despite the accumulation of pathology.

Building on over two decades of foundational work, we aim to fill in current conceptual gaps by (i) conceptualizing resilience as a continuum ranging from detrimental to beneficial, (ii) integrating genomic data to expand the search for further biological drivers of resilience, and (iii) quantifying our proteomic findings to maximize the utility of the resilience concept.

### Conceptualizing resilience as a continuum aligns it with many other risk factors

The dichotomous approach to the study of cognitive aging (dementia/no dementia, AD pathology/no AD pathology) has prevailed despite challenges to this approach presented by decades of epidemiological work (Morris, 2005; Howieson et al., 2008; Schneider et al., 2009; Sperling et al., 2011; Wilson et al., 2011; Bennett et al., 2012). Similarly, most studies have examined dichotomous resilience (resilient/not resilient) (Stern et al., 2023). Yet, if we regress out the negative effects of pathology, we can isolate cognitive decline due to pathologies from decline due to other (yet unknown) mechanisms.

**Figure 1:**
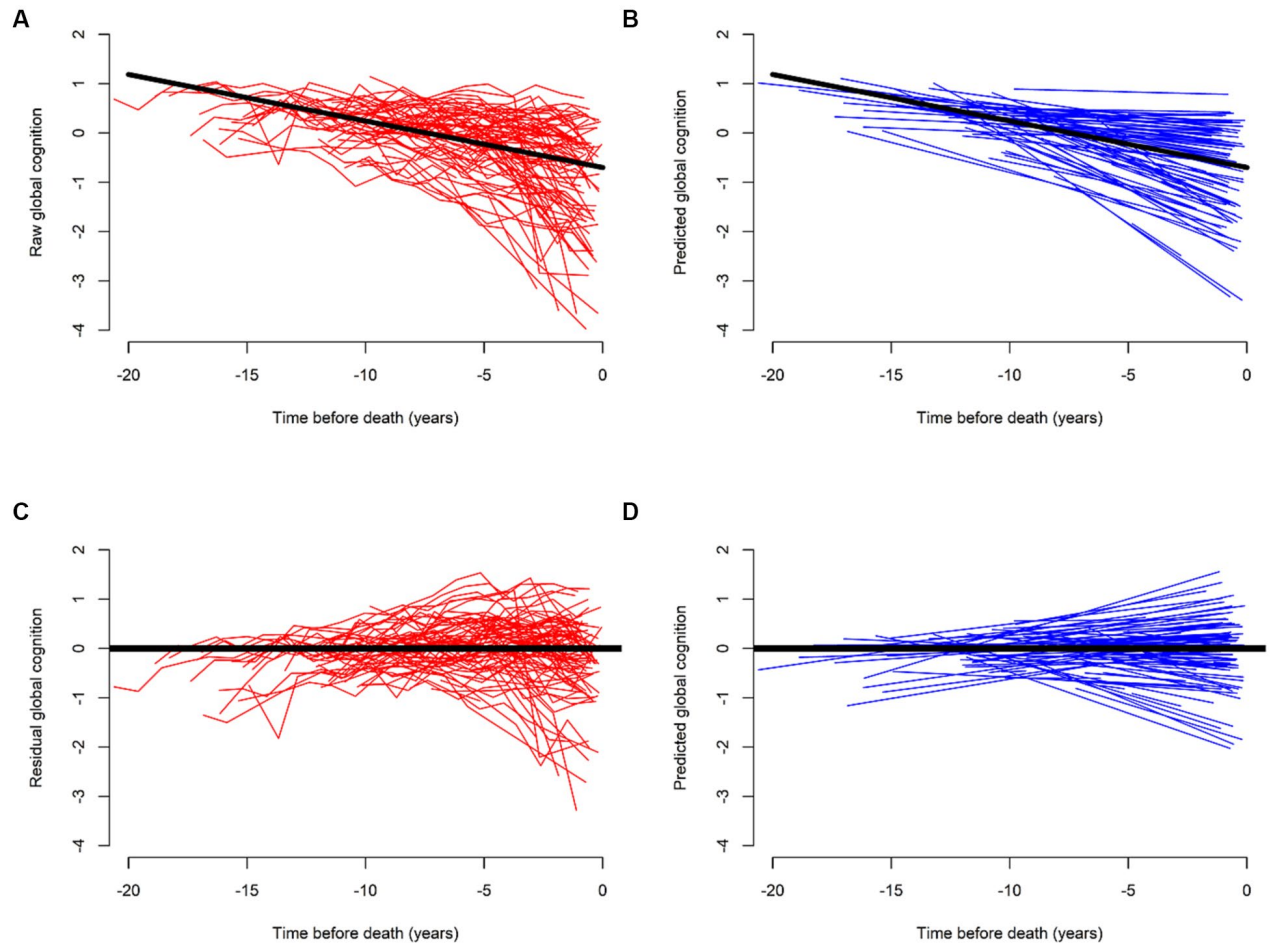
Trajectories of cognitive decline before (red) and after (blue) regressing out the effects of common brain pathologies. **(A,C)** Illustrate raw and **(B,D)** model-derived trajectories of cognitive decline for 562 participants. **(A,B)** Illustrate their trajectories without terms for brain pathologies. (**C,D)** Show reduced steepness of the slopes of cognitive decline as compared to **(A,B)**. This flattening, or improvement highlights the residual of cognitive decline after we regress out the effects of brain pathologies. Further modeling of residual cognitive decline with proteome can identify cognitive resilience proteins associated with cognitive decline that are unrelated to brain pathologies. We used data from ROS and MAP participants to generate this figure specifically for this perspective.

This residual approach, better operationalized as a positive (or negative) deviation from the regression line, reflects the average effect of the stressor (pathology) on the rate of cognitive decline. Each individual deviation from each observed outcome gives residuals of cognitive decline, ranging from negative (less resilience) to positive residuals (more resilience). Consequently, some individuals decline at slower rates than expected, while others decline at faster rates. We can see the raw trajectories and model-derived predicted slopes of cognitive decline before (Figures 1A,B) and after (Figures 1C,D) we regress out pathology. Any third factor that is associated with the residuals is a measure of either more or less resilience.

**Figure 2:**
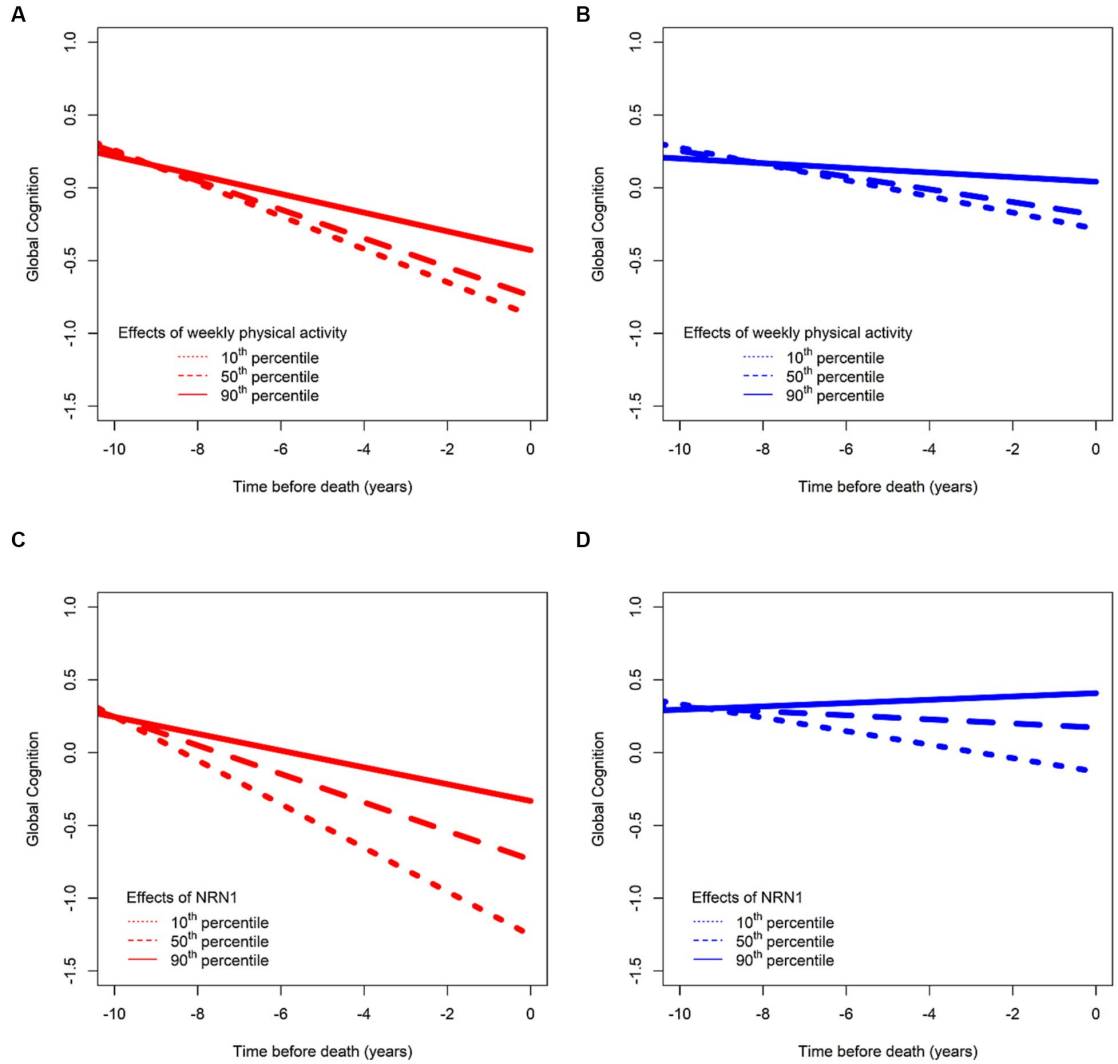
Effects of two risk factors on cognitive decline before (in red) and after (in blue) regressing out effects of common brain pathologies. Consistent with the concept that resilience is a continuous measure like other clinical risk factors, we illustrate here associations between total daily physical activities and cognitive decline in **(A,B)**. **(A)** The residual of cognitive function by regressing out age, sex, and education (depicted in red) for an average participant whose total daily physical activity is in the 10th (short dashed line), 50th (long dashed line), and 90th (solid line) percentiles and in **(B)** after further regressing out neuropathological indices (depicted in blue) for the same participant with total daily physical activity being in the 10th (short dashed line), 50th (long dashed line), and 90th (solid line) percentiles, with more activity being associated with higher cognition, and lower activity being associated with lower cognition. Similarly, like clinical risk factors, such as physical activity, we also illustrate the association between a biological resilience factor as a continuum (the protein NRN1) and its associations with cognitive decline after regressing out age, sex, and education **(C)** and after further regressing out neuropathological indices **(D)**, with a higher level of the proteins being associated with slower cognitive decline. We used data from ROS and MAP participants to generate this figure specifically for this perspective.

We previously identified a host of “third” factors associated with residual cognitive decline, that can be termed resilience factors. For instance, we found that higher daily physical activity, measured with a wrist-worn sensor, was associated with slower cognitive decline, and lower daily physical activity was associated with faster cognitive decline, even after we regressed out the negative effects of brain pathologies (Buchman et al., 2019). These data provide empiric evidence that daily physical activity may afford cognitive resilience unrelated to brain pathologies (Figures 2A,B).

We have also used biological markers, such as genes and proteins, to identify resilience mechanisms. For instance, Neuritin 1 (NRN1) is associated with slower cognitive decline independent of the effects of neuropathology (Yu et al., 2020; Zammit et al., 2023; Figures 2C,D). NRN1 is a neurotrophic factor, and a hub protein in a module associated with synaptic biology, and which plays an important role in synaptic function and plasticity (Naeve et al., 1997; Ng et al., 2023). Initially discovered as a neuronal activity-dependent synaptic gene product in the dente gyrus of the rat brain (Nedivi et al., 1993), NRN1 promotes axon regeneration by inducing neuritogenesis, arborization, and axonal elongation in the peripheral and central nervous systems (Shimada and Yamagata, 2013; Zhou and Zhou, 2014). The role of NRN1 in the formation of axonal arbors and dendritic branching in regulating neurodevelopment has been extensively reported. It has been associated with cognitive resilience in multiple studies in that higher abundance is associated with slower cognitive decline (Wingo et al., 2019; Yu et al., 2020; Hurst et al., 2023; Zammit et al., 2023). It has also been directly associated with indices of AD pathology (Yu et al., 2020; Hurst et al., 2023) in that individuals with higher abundance of NRN1 have less AD pathology. Lastly, there is also evidence that NRN1 mediates the association between cognitive resilience and AD pathology (Hurst et al., 2023). Collective evidence supports the promise of NRN1 as a therapeutic target to enhance synaptic resilience mechanisms in preclinical AD (An et al., 2014; Choi et al., 2014; Hurst et al., 2023; Ng et al., 2023).

Conceptualizing cognitive resilience as a biologic continuum extends current frameworks (Stern et al., 2020, 2023) by going beyond the ability to maintain better-than-expected cognitive function in the presence of brain pathologies to encompass the full spectrum of cognitive resilience. This allows us to identify a larger range of potential modifiable resilience behaviors or lifestyles that are either associated with slower, or faster rates of cognitive decline, independent of brain pathologies (Bennett et al., 2006c; Wilson et al., 2006, 2007a, 2013, 2014; Boyle et al., 2008, 2012; Buchman et al., 2019). This broader conceptualization of resilience provides more statistical power and more granularity than a dichotomous approach (Yu et al., 2020; Zammit et al., 2022). Further work is needed to identify which resilience lifestyle factors can be modified, or whether combinations of different behaviors can serve as a clinical signature or biomarker, identifying vulnerable older adults with lower-than-average resilience.

While the approach has also been criticized mainly due to the possibility that the residual measure might be correlated with the outcome (Elman et al., 2022), such a scenario would allow us to hypothesize possible unexplained variance between the error and the outcome. It might also be indicative of uncaptured non-linear decline. Despite critiques, the residual approach seems to capture clinical meaningful information about aging, cognitive decline, and the risk of dementia (Bocancea et al., 2021). Alternative solutions to the residual approach have been proposed (Elman et al., 2022), such as testing for effect-modification; however, we use these alternative approaches as validation steps, as we discuss further below. Analytic implementation of the residual method to resilience are likely to continue evolving with technological advancements in statistical modeling.

### Identifying biological mechanisms underlying cognitive resilience provides potential therapeutic targets

Discovering genes and proteins widely distributed in the central nervous system, that are likely candidates for the molecular mechanisms underlying cognitive resilience is a crucial first step for drug discovery studies for resilience therapies. Over the past decade, our group and others have applied our operationalization of resilience to different streams of genomic data to advance our understanding of its underlying biology (Wilson et al., 2013; Buchman et al., 2016; Mostafavi et al., 2018; Yu et al., 2018, 2020; Wingo et al., 2019). For instance, in prior work utilizing proteomic data (Wingo et al., 2019), 38 hub proteins associated with cognitive stability after adjusting for pathology were identified; proteins associated with more stability were enriched for higher synaptic function and more mitochondrial activity, while those associated with less stability (more rapid decline) were enriched for inflammatory response, apotosis, and endothelial function in glial cells. Co-expression network analysis of these hub proteins showed that largest modules with strongly expressed proteins relating to more cognitive stability were enriched for synaptic abundance and mitochondrial activity higher cognitive stability, while modules containing expressed proteins associated with less stability were enriched for apotosis, myelination, and inflammatory response. Overall, these findings lend support for subsequent work in ROSMAP cohorts that has aimed to identify cortical resilience proteins and explicate their underlying mechanisms.

In a recent proteome-wide study we examined the association of over 8,000 proteins from the dorsal lateral prefrontal cortex. We regressed out the effects of 10 ADRD pathologies on the slope of global cognitive decline. We identified 8 proteins (NRN1, ACTN4, EPHX4, RPH3A, SGTB, CPLX1, SH3GL1, UBA1) that remained associated with cognitive resilience (Yu et al., 2020). Consistent with the idea that resilience is a continuum, higher levels of 7 of these cognitive resilience proteins and a lower level of UBA1 were associated with a slower rate of cognitive decline. More recently we extended these results in a larger sample size and identified 47 proteins that are either commonly or specifically associated with five different cognitive abilities (Zammit et al., 2023). Supplementary Table S1 shows the full list of identified proteins. Gene ontology enrichment analyses showed that the majority of the resilience proteins associated with slower cognitive decline were enriched for mitochondrial and synaptic plasticity, which is supportive of prior work in other smaller cohorts which found similar pathways for proteins that are higher-abundance in cognitive stability independent of AD pathologic indices (Wingo et al., 2019). In addition, this work also provided evidence for both shared and distinct biological pathways associated with resilience in specific cognitive abilities. For instance, proteins associated with higher resilience in episodic memory were enriched for mitochondrial translation, while proteins associated with higher resilience in working memory were enriched in the prevention of the translation of mRNA into potentially harmful proteins (Zammit et al., 2023). Mitochondria are organelles that under the form of ATP molecules provide cellular energy for almost all processes from cell survival to death; in neurons, mitochondria are crucial for regulating and maintaining synaptic transmission, plasticity and neurotransmitter synthesis. Messenger RNAs on the other hand, are single-stranded molecules of RNA that are involved in the process of synthesizing proteins in the cytoplasm; they play a fundamental role in regulation of gene expression. Both enrichment of mitochondrial translation and regulation of mRNA translation have been implicated in AD (Liang et al., 2008; Reddy and Beal, 2008; Wingo et al., 2019; Ghosh et al., 2020; Rybak-Wolf and Plass, 2021); however, their differential role in specific cognitive abilities is largely unexplored and an area for potential future research.

More recently, we extended our proteomic studies of resilience to glycoproteomics. The study of glycoproteomics expands the potential to identify the mechanisms underlying cognitive resilience. A glycopeptiform is a protein that has one or more glycans attached to it. The addition of glycans to a protein is the most common post-translational modification in the brain. Glycan composition and its location on a protein can affect protein structure and function. Our recent glycoproteome-wide study of the DLPFC identified 8 glycopeptiforms associated with cognitive resilience; higher levels of four glycopeptiforms were associated with slower rates of cognitive decline and higher levels of another four were associated with a faster rate of cognitive decline (currently under review). These latter findings remained significant when controlling for the parent proteins. These findings suggest that glycopeptiforms derived from post-translational protein modification have a separate association with cognitive resilience after controlling for the parent protein. These results lend support that our residual approach can be extended to studies of other streams of genomic data to identify additional mechanisms underlying cognitive resilience in older adults.

### Resilience mechanisms can be quantified on a person-specific level

To leverage the potential therapeutic benefits of novel interventions or lifestyle changes that may afford resilience it will be necessary to develop instruments that can quantify some of the mechanisms driving resilience in the community or outpatient clinic settings so healthcare professionals can identify adults at risk of cognitive impairment to guide appropriate interventions. Efforts leveraging omics reviewed above highlight the need for an approach that can aggregate the varied effects of many molecular mechanisms, such as cortical proteins, likely to underlie cognitive resilience. In other clinical areas, risk scores have been used to aggregate the additive risks of multiple genes or clinical risk factors (Wolf et al., 1991; Desikan et al., 2017).

We have assessed the feasibility of developing a resilience index based on many resilience proteins that might be able to classify adults with higher- or lower-than-average resilience. We used a quantitative targeted protein pipeline (i.e., selective reaction monitoring) which enables large-scale, lower-cost measurement of 226 specific proteins, and leveraged data from 1,192 older decedents. We modeled cognitive decline using a global composite score and controlled for the effects of neuropathologic indices. We identified 52 proteins that were associated with residual cognitive decline, i.e., independent of the effects of neuropathologic indices. While 31 proteins were associated with less decline (more resilience), 21 were associated with steeper decline (less resilience) (Supplementary Figure S1A). A full list of the proteins can be found in Supplementary Table S2. We then aggregated the expression levels of these resilience proteins into a person-specific cognitive resilience index, allowing us to not only quantify resilience based on the expression of these proteins on a person-specific level but also to study it as a continuum, as discussed above (Supplementary Figure S1B). A higher level of the index was associated with a lower burden of postmortem AD pathology and was also associated with a 30% reduction in the detrimental effects of AD pathology on dementia proximate to death.

The cognitive resilience index constructed from many proteins was not specific for cognitive resilience and was also associated with other non-cognitive aging phenotypes including less decline in motor function and less severe parkinsonism. Supplementary Figure S2 highlights that while most of the proteins used to construct the cognitive resilience index are specific for cognitive resilience, about 30% of the proteins included in the cognitive resilience index also provide resilience to motor phenotypes. This emphasizes that resilience proteins can be pleiotropic and provide resilience for multiple phenotypes. This may explain the broad range of health benefits afforded by resilience factors.

**Figure 3:**
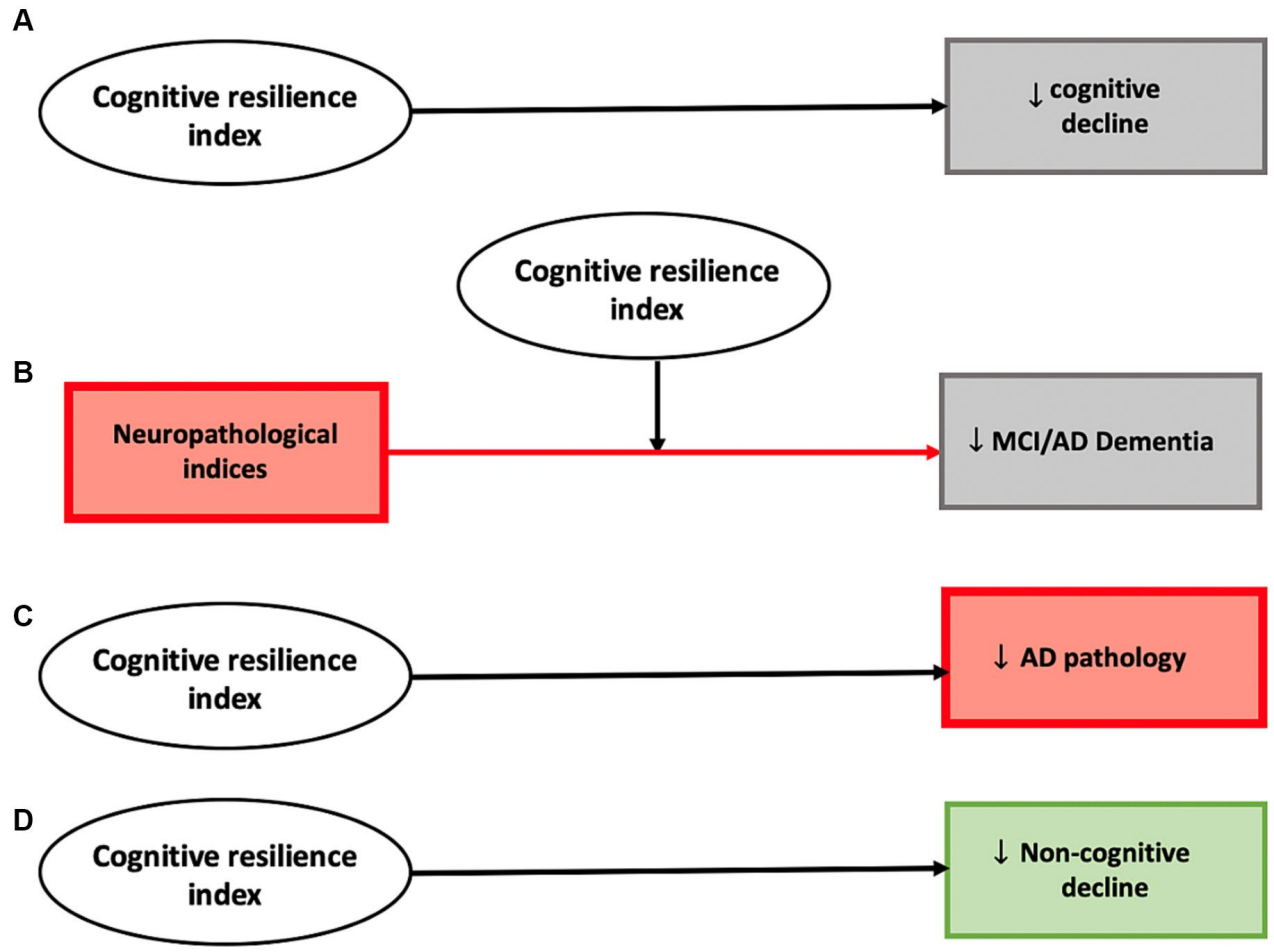
Analyses employing the constructed cognitive resilience index highlights several potential mechanisms through which cortical proteins can provide cognitive resilience. A higher cognitive resilience index derived from proteomic data demonstrated evidence for a direct negative association with cognitive decline **(A)**, modified the association between neuropathological indices and cognitive phenotypes including diagnoses of mild cognitive impairment and AD dementia **(B)**, was negatively associated with pathologic AD **(C)**, and was also negatively associated with decline in non-cognitive phenotypes (motor function and parkinsonism) **(D)**.

Using varied analytic approaches, these studies have identified three potential molecular mechanisms that can provide cognitive resilience. First, proteins can be associated with clinical traits independent of brain pathologies (identified using the residual approach). Second, proteins can interact with brain pathologies to attenuate or modify their negative association with clinical traits (using effect-modification). Third, proteins can directly affect the level of ADRD pathologies (also referred to as resistance in some studies; Bocancea et al., 2021). Our results for the constructed resilience index are summarized in Figure 3. These results suggest the feasibility of aggregating molecular drivers of resilience into a continuous index for the development of a clinical instrument that may be able to identify older adults at risk, who might benefit from resilience therapies to maintain cognition and other important aging traits. As the index was developed from omics obtained in decedents, this work will need to be translated into an index that can be employed in living adults to maintain cognition and brain health. While translational work is challenging, we recently developed AD imputation models, specifically trained to predict postmortem AD pathology in living adults by using clinical information up to 8 years before death (Yang et al., 2023). Such work can be extended to the resilience space. We also linked pathology and omic data to brain imaging and are currently extending that approach to the resilience space (Yu et al., 2017; Gaiteri et al., 2019; Makkinejad et al., 2021). Finally, we recently generated subpopulations with brain omic data and projected that to monocyte RNAseq data (Iturria-Medina et al., 2022). Overall, this is a challenge which we and others are trying to tackle to make brain omic data actionable in living humans.

### Resilience therapies may offer a novel approach to maintaining cognition in older adults

Advances in our understanding of brain pathologies have been crucial to the great strides that have been made over the past several decades in our understanding of the underlying biology of late-life cognitive impairment. Yet, despite these advances, nearly all brain pathologies remain untreatable. The studies reviewed above highlight the rapid advances in operationalizing the concept of resilience and explicating its underlying biology. Yet, more importantly, these advances may pave the way for new therapeutic options for late-life cognitive and physical impairments despite the lack of currently available treatments for nearly all brain pathologies.

Recent work suggests that the genetic architecture of resilience is distinct from that of AD dementia related to brain pathologies (Dumitrescu et al., 2020). Considering resilience, instead of brain pathologies, as a therapeutic target, offers a novel paradigm for maintaining cognition and brain health in aging adults via modifiable resilience behaviors and new resilience therapies. Using resilience as a therapeutic target may support cognition even in the presence of the negative cognitive effects of accumulating brain pathologies that are currently untreatable. Moreover, targeting resilience may even have a greater impact on cognitive outcomes than interventions targeting specific pathologies. Indeed, even if therapies are developed for a specific pathology (Mintun et al., 2021; van Dyck et al., 2023), the beneficial effect from treating a single pathology might be minor given the large number of combinations of person-specific burden of mixed-brain pathologies observed in aging brains (Boyle et al., 2018, 2021).

Given the challenges of highly heterogeneous neuropathologic stress and its varied negative effects on cognitive decline and brain health across individuals, it seems more plausible and advantageous to redirect efforts to potential therapeutic interventions unrelated to accumulating pathologies. Hence, advancing the initial studies done to date with further mechanistic and drug discovery studies has potential to catalyze the development of interventions focusing on resilience behaviors or novel drugs that can be deployed to maintain cognition and other vital aging phenotypes even in the presence of aging brains that accumulate untreatable mixed brain pathologies.

Extending the successful approaches employed in these initial studies to a wider range of phenotypes and to more brain regions and tissues outside the brain that support the distributed networks underlying non-cognitive aging phenotypes will expand the therapeutic toolkit available for resilience therapies and eventually lead to personalized and targeted resilience treatments.

## Conclusion

Conceptualizing resilience as a continuum aligns it with many other conventional risk factors. This conceptualization has been crucial in quantifying, and operationalizing studies of resilience using varied analytical approaches that regress out the negative cognitive effects of brain pathologies. Studies analyzing novel streams of genomic data have isolated cognitive resilience unrelated to brain pathologies to identify genes and proteins that degrade or enhance resilience. These varied molecular mechanisms lend additional empirical support for considering resilience as a continuum. All individuals may have some degree of resilience. Some individuals may have more genes and proteins that enhance resilience, yielding higher-than-average function, and some adults may have more genes and proteins that degrade resilience producing vulnerability and lower-than-average resilience. While there are thousands of genes and proteins in the human brain, the molecular mechanisms identified in recent resilience studies provide high-value therapeutic targets for further mechanistic and drug discovery studies that can catalyze new resilience therapies that maintain cognition and brain health in aging adults even in the presence of untreatable brain pathologies.

## Data availability statement

The original contributions presented in the study are included in the article/Supplementary material, further inquiries can be directed to the corresponding author.

## Ethics statement

The studies involving humans were approved by the Rush Institutional Review Board. The studies were conducted in accordance with the local legislation and institutional requirements. The participants provided their written informed consent to participate in this study.

## Author contributions

AZ: Writing – original draft. DB: Writing – review & editing. AB: Writing – review & editing.
