# Supplementary material for: From theory to practice: translating the concept of cognitive resilience to novel therapeutic targets that maintain cognition in aging adults
Source: Front Aging Neurosci. 2024 Jan 12;15:1303912. doi: 10.3389/fnagi.2023.1303912 (PMC10811007; doi:10.3389/fnagi.2023.1303912)
Supplement: Supplementary file 1 [file Table_1.docx]

**Supplementary files**

**Supplementary Table 1.** Full list of proteins associated with resilience across different cognitive abilities identified using TMT proteomics in the ROSMAP cohorts^63^.

| **Global cognition** | | **Working memory** | | **Episodic memory** | | **Semantic memory** | **Processing speed** |
| --- | --- | --- | --- | --- | --- | --- | --- |
| ++ | -- | ++ | -- | ++ | -- | ++ | -- |
| NRN1 | PAFAH1B3 | NRN1 | PAFAH1B3 | NRN1 | PAFAH1B3 | NRN1 | CHGA |
| NT5DC1 | PALM3 | NT5DC1 | PALM3 | NDRG2 | PALM3 | TLN |  |
| ADAM11 | TMEM30A | ADAM11 | TMEM30A | AHCYL1 | TMEM30A | TMEM141 |  |
| MAGEA8 | CHGA | MAGEA8 | CHGA | TLN | UBA1 | CADPS2 |  |
| CKAP4 | EEF1A2 | CKAP4 | EEF1A2 | CKAP4 | CLCN3 |  |  |
| SLC8A3 | RPS6KA2 | SLC8A3 | RPS6KA2 | PRM1 | SLC5A3 |  |  |
| NDRG2 | AMPD2 |  | AMPD2 |  | TBXA2R |  |  |
| AHCYL1 | ADGRG1 |  | ADGRG1 |  | EFF1A2 |  |  |
| TLN | MACROD1 |  | MACROD1 |  |  |  |  |
| PRM1 | L1CAM |  | L1CAM |  |  |  |  |
| CYB5R1 | SESN1 |  | SESN1 |  |  |  |  |
| ACTN4 | SLC5A3 |  | SLC5A3 |  |  |  |  |
| MIGA1 | TBXA2R |  | TBXA2R |  |  |  |  |
| TIMM8B | UBA1 |  | PSMB1 |  |  |  |  |
| GOSR1 | CLCN3 |  | CLD10 |  |  |  |  |
| RPS15A | SNX12 |  | TMEM245 |  |  |  |  |
|  | ABCC5 |  |  |  |  |  |  |
|  | UPF2 |  |  |  |  |  |  |
|  | SERPINB11 |  |  |  |  |  |  |
|  | RAB6A |  |  |  |  |  |  |
|  | UPF3B |  |  |  |  |  |  |
|  | CNST |  |  |  |  |  |  |
|  | GABARAPL2 |  |  |  |  |  |  |
|  | MAP2K6 |  |  |  |  |  |  |
|  | RGS17 |  |  |  |  |  |  |
|  | ATP8A1 |  |  |  |  |  |  |

*Note. Proteins in the ++ column depict positive associations with cognitive resilience i.e. slower rate of cognitive decline, and proteins in the - - column depict negative associations with cognitive resilience i.e. faster rate of cognitive decline. There were no proteins negatively associated with semantic memory, and there were no proteins positively associated with processing speed. There were no associations with visuospatial ability.*

| **Global cognition** | |
| --- | --- |
| **++** | **--** |
| VGF_1 | tau_12E8_s262 |
| VGF_2 | IGFBP5_1 |
| SYT12_1 | tau_AT100_t217 |
| STX1A_1 | AMPD2_2 |
| CD47_1 | GSTP1_1 |
| NDUFA7_2 | CLU_2 |
| NDUFS6_1 | PTMS_1 |
| tau_AT8 | HSPB2_2 |
| SNAP25_2 | C9orf16_2 |
| NDUFA7_1 | AK4_2 |
| STXBP1_6 | RUVBL1_2 |
| SNAP25_1 | FYN_3 |
| NDUFA10_2 | TPRG1L_1 |
| tau_AT100 | tau_77G7_s305 |
| ITPK1_1 | AK4_1 |
| SYT7_2 | SLC6A12_1 |
| NDUFS6_2 | VAMP5_1 |
| SNAP25_4 | VAT1_2 |
| UQCR10_2 | SYN2_2 |
| STX1A_2 | GRB2_2 |
| CD47_2 | GFAP_1 |
| NDUFA6_2 |  |
| NDUFV1_2 |  |
| UQCRC2_2 |  |
| UQCRC2_1 |  |
| VAPB_2 |  |
| ND UFA5_1 |  |
| SNAP25_3 |  |
| BIN1_4 |  |
| SNAP25_6 |  |
| NDUFV1_1 |  |

**Supplementary Table 2.** List of the 52 proteins associated with resilience across in global cognition identified using SRM proteomics in the ROSMAP cohorts^70^.

*Note. Proteins in the ++ column depict positive associations with cognitive resilience i.e. slower rate of cognitive decline, and proteins in the - - column depict negative associations with cognitive resilience i.e. faster rate of cognitive decline.*

**
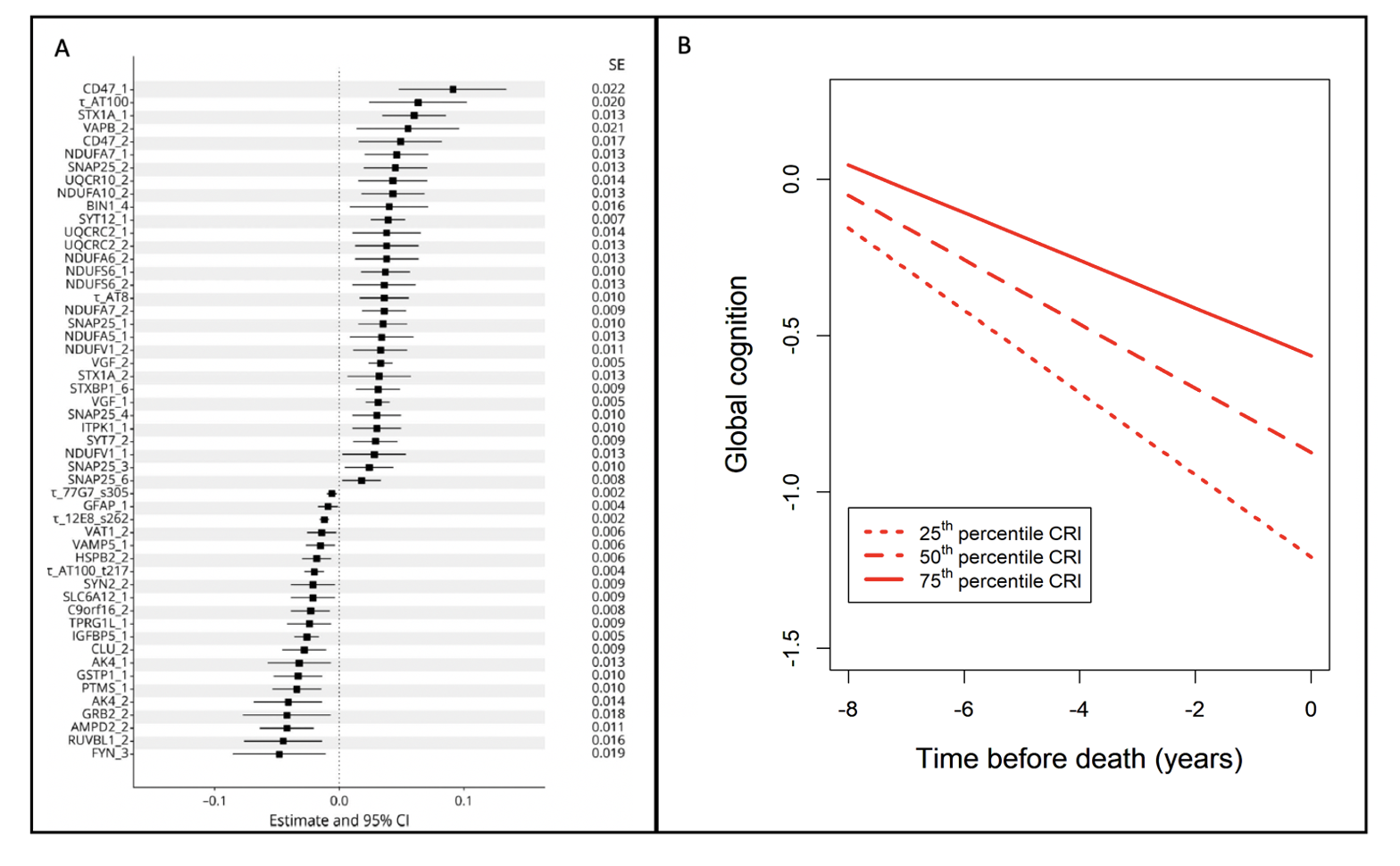
**

**Supplementary Figure 1. The proteomic-derived cognitive resilience index as a continuum.** The effects of the 52 proteins on cognition can be seen in the forest plot (panel A). As can be seen in plot, higher levels of 31 of the proteins and lower levels of 21 were associated with higher resilience (i.e. slower cognitive decline). Note that none of the proteins provide zero resilience, but are rather spread out across a continuum providing lower (left of zero) or higher (right of zero) resilience. When we aggregate the 52 proteins on a person-specific level, we can plot empirically derived slopes modelling cognitive resilience on a continuum with any three 90^th^ year old individuals functioning at the 25^th^, 50^th^, or 75^th^ percentiles (Panel B). These slopes reflect cognitive resilience based on the aggregation of 52 cortical resilience proteins, that were associated with cognitive decline independent of the effects of pathology. All proteins were associated with cognitive decline at FDR-adjusted *p* <0.05. Figures adapted from Zammit et al. ^70^


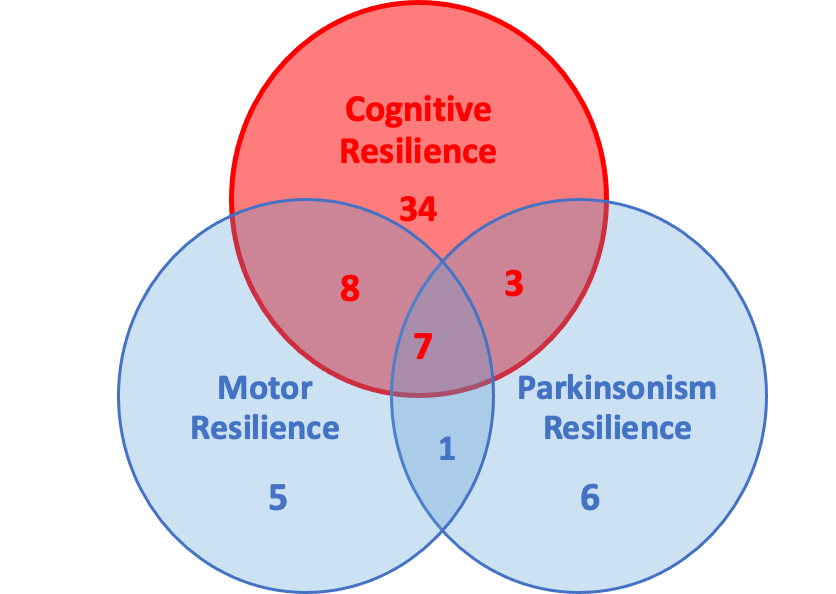


**Supplementary Figure 2. Some cortical proteins may provide resilience for several aging phenotypes and some may provide resilience for specific aging phenotypes.** This figure shows that out of 226 cortical proteins, 52 were associated with cognitive resilience (bolded in red), 34 of which were specific to cognition, 8 of which were also associated with motor resilience, 3 of which were associated with parkinsonism resilience, and 7 of which were associated with both motor and parkinsonism resilience. Of the 226 proteins, we also found that 5 proteins were specifically associated with motor resilience, 6 specifically associated with parkinsonism resilience, and 1 with both. These results indicate the pleiotropy of some of these proteins and the specificity of others for different phenotypes. Figure adapted from Zammit et al.^70^
